# Acromegaly and male sexual health

**DOI:** 10.1007/s11154-022-09721-0

**Published:** 2022-04-01

**Authors:** Gianmaria Salvio, Marianna Martino, Giancarlo Balercia, Giorgio Arnaldi

**Affiliations:** Division of Endocrinology, Department of Clinical and Molecular Sciences, Polytechnic University of Marche, Umberto I Hospital, Via Conca 71, 60126 Ancona, Italy

**Keywords:** Growth hormone, Erectile dysfunction, Prostate cancer, Hypogonadism, Testicular volume

## Abstract

Acromegaly is a rare pathology characterized by chronic hypersecretion of Growth Hormone (GH) and Insulin-like Growth Factor-1 (IGF-1) that causes somatic, metabolic, and systemic changes. The somatotropic axis acts physiologically favoring gonadal function, but when GH is produced in excess it has deleterious effects on many aspects of male sexuality. It is widely demonstrated, in fact, that acromegaly induces hypogonadism through different mechanisms, both through direct mass effect on gonadotropic cells and through increased plasma levels of prolactin. Moreover, hypogonadism is also one of the factors linking acromegaly to erectile dysfunction (ED), but also metabolic complications of acromegaly and, probably, GH itself contribute to the genesis of this disorder. There are few data in the literature on the impact of the disease on fertility and testicular volume. Finally, knowledge of the role of GH hypersecretion on the occurrence of prostatic diseases such as benign prostatic hypertrophy and prostatic cancer appears to be of fundamental clinical importance in the long-term management of these patients.

## Introduction

Acromegaly is a rare systemic pathology (incidence 2–11 cases/million/year) characterized by a chronic hypersecretion of Growth Hormone (GH) and consequently of its peripheral "effector" Insulin-like Growth Factor-1 (IGF-1). This disease causes somatic changes and metabolic and systemic alterations; if not treated or controlled by therapy, it’s associated with increased mortality mainly due to cardiovascular and neoplastic causes [1].

Due to the slow and chronic course of the disease, diagnosis often occurs several years after the appearance of the first symptoms, when complications are already present. Considering the close correlation that exists physiologically between somatotropic axis and gonadal function, it is not surprising that the andrological component is also altered in acromegaly, which affects about half of male subjects.

The present review summarizes the available evidence and clinical aspects of this important side of a person's life.

## Somatotropic axis and gonadal function: physiological aspects

**Fig. 1:**
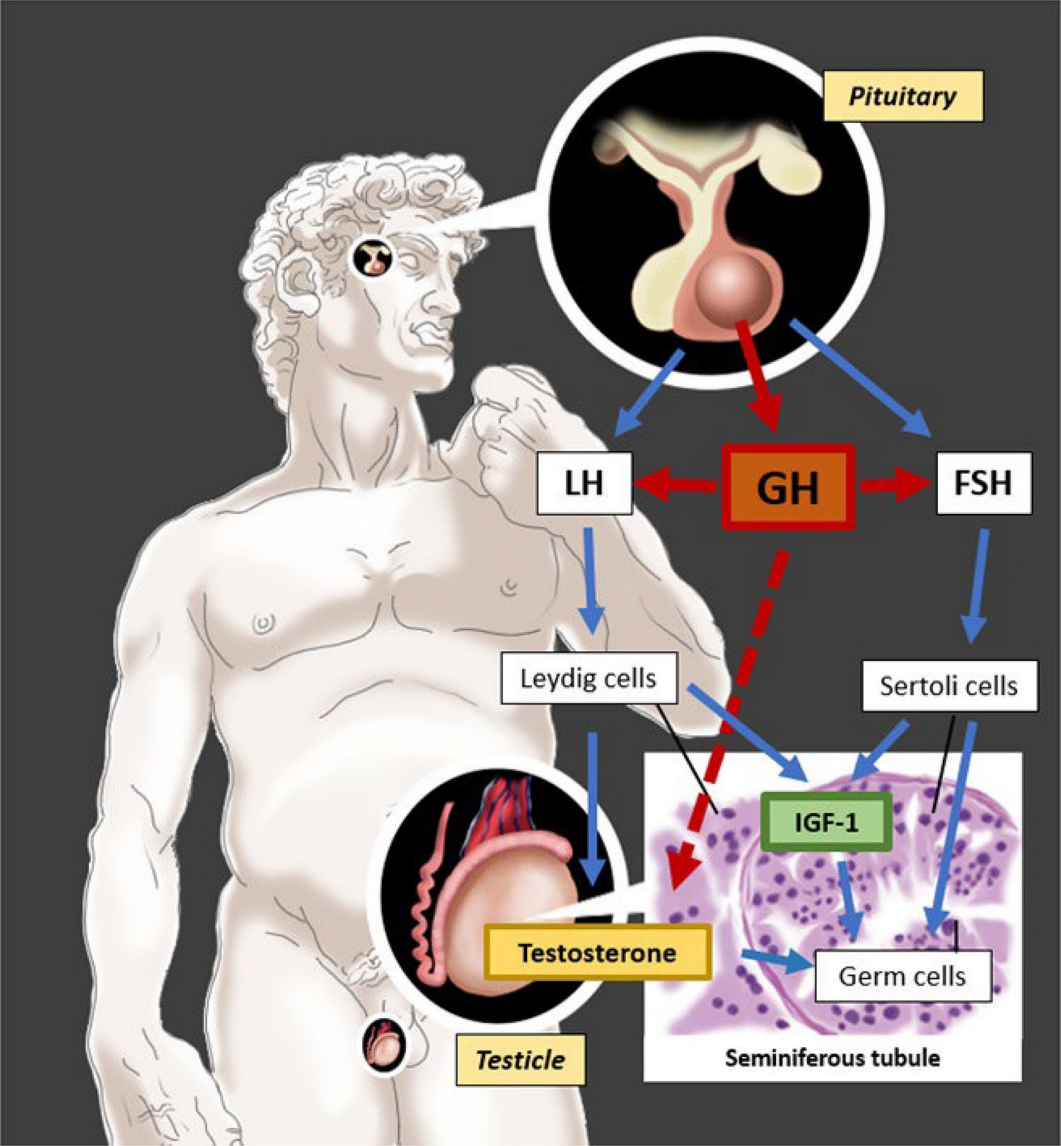
**Somatotropic axis and gonadal function.** Excess Growth hormone (GH) exerts an inhibitory effect on the release of gonadotropins (Luteinizing Hormone, LH, and Follicle Stimulating Hormone, FSH). Reduced LH is accompanied by reduced stimulation of testicular Leydig cells, resulting in the development of hypogonadism. Reduced FSH, on the other hand, is accompanied by reduced activity of Sertoli cells, which act as a support for germ cells, resulting in reduced fertility. On the other hand, Insulin-like Growth Factor-1 (IGF-1) is produced physiologically by both Sertoli and Leydig cells supporting both spermatogenesis and endocrine function of the testis

There is a close correlation between somatotropic axis and gonadal function (Fig. 1). The two axes, since the early stages of growth, play a leading role in a two-way dialogue through which GH, IGF-1 and sex steroids guide the pubertal development. In the pre- and peripuberal age, in fact, a marked increase in GH secretion has been observed in response to the administration of exogenous testosterone, both at physiological and pharmacological doses, probably due to aromatization to estrogen. Similarly, in elderly and hypogonadic subjects, a GH increase can be detected in response to the administration of testosterone or Gonadotropin-Releasing Hormone (GnRH). Curiously, the estrogenic state, in female subjects, does not seem to show the same positive correlation [2] despite GH exerts an important role in the regulation of ovulation and fertility. On the other hand, studies in animals and humans have shown the existence of an intra-testicular production of IGF-1 by Sertoli and Leydig cells, which would contribute to spermatogenesis and endocrine function of the testicle. In the detail, IGF-1 is released by both Sertoli and Leydig cells under gonadotropin regulation and plays an autocrine and paracrine role on both testicular steroidogenesis and spermatogenesis, as supported by the identification of IGF-1 binding sites in Leydig and Sertoli cells and primary spermatocytes [3]. As a demonstration of this, in animal models, the absence of IGF-1 prevents the maturation of Leydig cells, compromising their steroidogenic capacity [4]. In humans, Laron's Syndrome provides evidence to support the transability of this observation in our species: individuals affected by this disease do not produce IGF-1 and experience a deficient development of reproductive function and growth retardation, both corrected by administration of exogenous IGF-1 [5]. Similarly, in hypophysectomized rats, GH administration increases the content of receptors for Luteinizing Hormone (LH), increasing the responsiveness of the testis to gonadotropin administration [3].

A further demonstration of this close link has also been histologically detected at pituitary level: pituitary cells expressing mRNA for Follicle-Stimulating Hormone (FSH) and LH or receptors for GnRH showed a positivity in immunohistochemistry for GH, suggesting a possible mixed function of these cells (somatotropic and, transiently, gonadotropic) or a co-regulation of two different hormonal axes by the same population of pituitary cells [3].

Taken together, these data underscore the close link between somatotropic and gonadal axes, leading to the hypothesis that dysregulation of the former may have profound effects on the latter. In acromegaly, some effects on male sexuality, in fact, appear to be closely related to the increase of GH and IGF-1 acting both at hypothalamic-pituitary and testicular level (see further in the text), while others present a more complex pathophysiological mechanism in which additional factors are involved. In the following sections these aspects will be explored in detail, to provide an overall account of the effect of acromegaly on the sexual health of men.

## Acromegaly and hypogonadism

Hypogonadism is a frequent endocrine manifestation directly or indirectly related to acromegaly, affecting from 30 to 50% of these patients [6, 7]. In fact, the presence of a pituitary macroadenoma (which may be present in 77% of subjects with acromegaly) can inhibit the gonadotropic function by direct compression of gonadotropic cells or causing a pituitary stalk deviation with consequent hyperprolactinemia. It is not uncommon that the latter may derive directly from a cosecretion by the adenoma itself. Regardless of the size of the adenoma, however, the high levels of GH and IGF-1 act at the hypothalamic-pituitary level altering the regular pulsatility of the gonadotropin secretion and resulting in a state of hypogonadotropic hypogonadism. Based on this premise, a Consensus of experts recommends measurement of total testosterone, Sex-Hormone Binding Globulin (SHBG) and prolactin for the evaluation of gonadal function in men with acromegaly at diagnosis and then annually [6]. Finally, a picture of hypogonadotropic hypogonadism (isolated or associated with other hormonal defects) may also be the consequence of surgical or radiant treatment [1]. According to a very recent report, hypopituitarism is a common complication of surgery for pituitary adenomas, affecting up to one-third of patients within three months from the operation [8], as confirmed by do Amaral et al., who found similar rates both for primary and for revision surgeries [9] Interestingly, Raikundalia et al. recently reported a lower frequency of iatrogenic hypopituitarism in patients with acromegaly undergoing transsphenoidal surgery than in non-acromegalic subjects, although this finding demonstrated only borderline significance on multivariate analysis [10]. After Gamma Knife radiosurgery, a slightly lower incidence of hypopituitarism has been reported, but the delayed time of onset implies the need for careful follow-up over time [11].

Among the typical features of hypogonadism such as decreased libido end Erectile Dysfunction (ED), gynecomastia could arise in acromegaly due to the stimulation of GH and IGF-1 on the mammary gland, as demonstrated on aging primates [12]. Despite this, reports in humans have not shown this abnormality except in association with incident hypogonadism [13] further studies are needed, since there are no clear data on a possible increased incidence of gynecomastia or worsening of other symptoms of hypogonadism, such as low libido or decreased morning/nocturnal erection in patients with acromegaly.

## Acromegaly and erectile dysfunction

ED is a common finding in general population, especially in presence of multiple cardiovascular risk factors, and its prevalence is particularly high in patients with acromegaly, ranging from 42 to 60% [14, 15]. Even if well recognized, the association between acromegaly and ED is far from being fully understood and several hypotheses have been advanced. Hypogonadism and metabolic complications of acromegaly (diabetes, hypertension, cardiomyopathy and obstructive sleep apnea syndrome) [16] are thought to act synergistically hindering the regular sexual function of these subjects. Although a related-disease psychological effect is possible, data collected using the Structured Interview on Erectile Dysfunction (SIEDY) for pathogenic quantification has highlighted the dominance of the organic component in the genesis of the dysfunction [15]. As in the general population, therefore, the presence of ED in patients with acromegaly allows to highlight a subgroup of subjects with a more marked dysmetabolic component who deserve a closer follow-up and a more careful control of cardiovascular risk factors [17]. This is what came out from a retrospective study by Lotti et al. that analyzed a group of 57 acromegalics, comparing the different characteristics of subjects with ED (n = 24) and without ED (n = 33). The ultrasound and clinical data (the latter collected also with the help of validated questionnaires—SIEDY and Middlesex Hospital Questionnaire) showed a greater prevalence of metabolic complications among those with ED, with a consequent increased incidence of major cardiovascular events. A further comparison was then made between acromegalic patients with ED and "healthy" subjects with ED. Subjects with acromegaly and ED had a significantly worse ED problem (and a higher organic component of ED, according to SIEDY), a higher prevalence of prior major cardiovascular events and worse results at penile doppler ultrasound evaluation [15]. In line with these findings, a direct effect of GH hypersecretion on erectile function has also been suggested. In fact, a recent study has shown that GH levels directly correlated to the severity of ED in 51 males with acromegaly and that Nitric Oxide (NO) levels were significantly lower in acromegalic patients than in non-acromegalic controls [14], supporting the hypothesis of acromegaly as a direct cause of endothelial dysfunction, as previously suggested by other authors [18]. Moreover, Chen et al. reported higher random GH levels and higher nadir levels of GH after Oral Glucose Tolerance Test (OGTT) in patients with ED, but, surprisingly, no significant association was found between ED and testosterone, BMI, diabetes, hypertension, or previous coronary artery disease [14].

The perception of bodily appearance, moreover, could negatively affect the search for intimacy and physical contact. Subjects with acromegaly, in fact, have a negative body image that is accompanied by intimacy problems and low affiliation [19] and that is further worsened by the presence of depressive symptoms [20]. Given the bidirectionality between depression and ED [21], it goes without saying, therefore, that acromegaly could feed a visual circle among these two conditions.

Taken together, these data clearly indicate that ED is a common manifestation in patients with acromegaly, in whom it occurs with greater severity than in the general population. GH excess per se could represent a key element in the pathogenesis of this disorder, but little evidence is available. In addition, no one, to the best of our knowledge, has evaluated whether the control of acromegaly is able to improve erectile function or the efficacy of classic treatment for ED (e.g., phosphodiesterase 5 inhibitors) in patients with acromegaly.

Given the increased cardiovascular morbidity independently associated with either acromegaly or ED, it goes without saying that adequate risk stratification in patients with acromegaly should already include assessment of routine sexual function and future research should address these aspects of acromegalic pathology.

## Acromegaly and male fertility

Although gonadal function disorders are a very frequent problem in acromegaly patients, few authors have focused their attention on complications in terms of male fertility.

Until a short time ago, the only available study was the one of Colao et al. [22], in a population of 35 subjects with newly diagnosed acromegaly aged 29–59 years after 6 months of treatment with first-line therapy (medical or surgical). After treatment, all patients showed a significant increase in testosterone levels, while only in subjects with controlled disease there was an increase in FSH and LH levels. Similarly, all patients showed an increase in spermatozoa, while motility was significantly increased only in subjects with controlled disease. The treatment of acromegaly, with spontaneous correction of hypogonadism and reduction of prostate inflammation [23], appears to be the fundamental element for the recovery (or the preservation) of fertility. The authors also argue that, if the recovery of the gonadal axis does not occur spontaneously within six months of treatment, androgenic replacement therapy with gonadotropins or testosterone, depending on whether paternity is desired or not, should be considered to prevent consequences of hypogonadal state [22]. In contrast to these observations, a very recent study found that although acromegalic patients have drastically reduced testosterone levels, they show no differences in terms of seminal volume, sperm count and sperm motility compared to healthy subjects [24]. As reported above in the text, somatotropic and gonadotropic axis find a meeting point in the testis, where GH and IGF-1 exert permissive effects on Leydig and Sertoli cells [3–5]. Moreover, animal models have shown that exogenous GH and IGF-1 can improve the motility and morphology of immature spermatozoa [25]. This has led to hypothesize a possible role for exogenous GH in promoting spermatogenesis in both patients with idiopathic infertility and those with GH Deficiency (GHD), but results were conflicting. Some authors reported a positive effect of GH treatment both in GHD [26, 27] and in non-GHD [28, 29] patients, whereas others have found no substantial changes in seminal quality [30, 31]. Further, larger, well-designed studies are therefore needed to reach any conclusions. Moreover, despite the changes in the quality of seminal fluid reported so far, no data are available regarding the risk of abortion and/or fetal malformations in the offspring of men with acromegaly and this is also a critical point to be explored in depth.

## Acromegaly and testicular volume

There are no studies in the literature, at the moment, that have gone to compare the size of testicles in patients with acromegaly compared to the general population, despite a recent review by Cannarella et al. [32] emphasizes the close connection between the somatotropic axis and the testicle. In particular, IGF-1 assumes a permissive role towards FSH and directly influences the development and proliferation of germ cells and Sertoli cells, which, from the volumetric point of view, represent the major component in the testicular parenchyma. In 2004 a case of seminoma accidentally discovered in a 26-year-old boy with bilateral macrorchidism was reported. The boy came to clinical attention on suspicion of acromegaly on the basis of signs of hypogonadism associated with swelling of the extremities. The confirmed acromegaly was due to pituitary macroadenoma with hypogonadotropic hypogonadism and hyperprolactinemia due to pituitary stalk deviation. The interesting observation was that, apart from the testicle affected by seminoma, where the growing lesion had determined an obvious volumetric increase of the organ, the contralateral testicle also appeared significantly enlarged (>30 ml), despite the reduced levels of FSH. The surgical cure of acromegaly, with consequent return of hypogonadism and increase in FSH, was "paradoxically" accompanied by a volumetric reduction of the testicle [33]. This data would therefore suggest a direct effect of GH on testicular volume, although further clinical confirmation is required. At the opposite extreme, the case of the simultaneous presence of acromegaly and testicular atrophy (volume < 2 ml) in a 27-year-old boy was recently reported. The presence of hypergonadotropic hypogonadism and the exclusion of a gonadotropinoma by immunohistochemical investigation of the surgical specimen after transsphenoidal surgery suggested the diagnosis, subsequently confirmed, of a rare association between acromegaly and Klinefelter syndrome [34]. In this case, it is likely that the severe genetically determined testicular impairment outweighed a possible trophic effect of GH, although this remains only speculative.In this context, a very peculiar pathological situation is represented by McCune-Albright syndrome, a genetically determined condition characterized by fibrous mono/poliostotic dysplasia, café-au-lait spots and endocrinopathies due to hyperfunction. In about 20–30% of cases, subjects present acromegaly [35], while up to 50% of males have macrorchidism, but, despite the increase in testicular volume, the development of precocious puberty supported by an effective production of testosterone occurs in a minority of patients, i.e. those in whom Leydig cells are hyperfunctional [36]. In other cases, the volumetric increase of the testicle would derive from the activation of the Sertoli cells only, whose proliferation may represent the manifestation of the mutation of the GNAS gene, typical of the syndrome. However, for those subjects who develop acromegaly in the context of McCune-Albright syndrome, a participation by the GH-IGF-1 axis cannot be completely excluded. Further studies are therefore necessary, especially since data on the consequences of McCune-Albright syndrome on male gonadal function and fertility are scarce.

## Acromegaly and prostatic hypertrophy

Although testosterone and dihydrotestosterone are the major regulators of prostate trophism, recent evidence has shown that, although essential, they are not the only factors involved [37]. In this regard, a possible influence of GH and IGF-1 on the growth of the prostate gland has been recently suggested.

The visceromegaly is a well-known complication of acromegaly [1]. However, if we consider the hypogonadotropic hypogonadism in these patients [38], we may expect that the reduction of circulating androgen levels could represent a protection against the development of prostatic pathologies [39]. In this regard, Colao et al. evaluated, by transrectal ultrasound, the prostate volume before and after one year of medical therapy in a group of 10 newly diagnosed acromegaly subjects. Surprisingly, despite the young age of the subjects (26–39 years) and the presence, in all of them, of secondary hypogonadism, the average prostate volume at the beginning of the study was very high (29.8 ml versus 18.2 ml in healthy subjects of the same age), and 3 patients presented frank prostatic hypertrophy (volume > 30 ml). After treatment with octreotide in association with cabergoline in patients with coexisting hyperprolactinemia, the mean prostate volume showed a significant reduction (22.1 ml), despite a simultaneous increase in testosterone levels to complete normalization [40]. The authors subsequently extended the evaluation to 46 acromegalic patients (26–74 years), reporting not only a close correlation between the presence of prostatic hypertrophy and the degree of disease activity, but even that the prostate volume appears to be conditioned, even more than by age, by the duration of the disease. Compared with "healthy" subjects, moreover, acromegalic patients have a threefold increased prevalence of sonographic abnormalities such as calcifications and cysts [41]. The disease control leads to a reduction in prostate volume, regardless of the mode of treatment as confirmed by another study conducted on 23 acromegalics (29–70 years). This should be due to direct mitogenic effect of GH and IGF-1 on the prostate and highlights the reversibility of this effect, which is not linked to a specific antiproliferative action of somatostatin analogues (as it has been previously hypothesized) [42]. Interestingly, no data currently exist on the efficacy of standard treatments for prostatic hypertrophy in individuals with acromegaly.

In 2013, Correa et al. confirmed a very high prevalence of prostatic hypertrophy in 40 acromegalics over 40 years of age (46.15%, compared to 12.50% of healthy controls) and a significant reduction in the average prostate volume after only one year of therapy (25.50 ml compared to 28.50 ml baseline) [43]. Finally, Kumar et al. also reported an increased frequency of ultrasound alterations and increased prostate volume in subjects with acromegaly, with a greater prevalence of obstructive urological symptoms. However, given the low clinical impact of the detected alterations, authors questioned about the usefulness of a routine prostate evaluation in this category of subjects [44], an argument that is still a matter of debate, so much so that in the Consensus of experts dedicated to the management of complications, prostate evaluation is not included among the systematic evaluations [6].

## Acromegaly and prostate cancer

Although GH and IGF-1 are implicated in carcinogenesis, as supported by epidemiological and experimental data [45], the increased risk of cancer in acromegaly remains an unclear issue.

The latest Italian multicentric study confirms a moderate increase in the incidence of neoplastic diseases in acromegalics, especially in districts such as colon, kidney and thyroid [46], with an associated increase in cancer-related mortality [47]. However, at least in these studies, no prostate cancer data are available. A very recent nationwide cohort study (1978 to 2010) including 529 acromegaly patients showed a standardized incidence ratio (SIR) of 1.4 for colorectal cancer, 1.1 for breast cancer, and 1.4 for prostate cancer. In the same study, a meta-analysis of cancer SIRs from 23 studies was performed. Even if the authors excluded from the analysis all tumors discovered during the first year of diagnosis in order to avoid a potential bias of surveillance, they reported an increased incidence of neoplasms with an SIR of 1.5. Specifically, acromegalic subjects would have a slightly higher incidence of prostate cancer than the general population, with an SIR of 1.2, even not statistically significant [48]. It is also true that, compared to other types of tumors, including colorectal cancer, prostate neoplasia very often presents indolent behavior, with very long onset and progression times [49]. Therefore, studies capable of detecting its incidence necessarily require extremely long observation times and, moreover, no studies have systematically investigated this category of subjects with targeted invasive evaluations (e.g., prostate-specific antigen and prostate biopsy), so it can be expected that the actual incidence of prostate cancer has been underestimated. Therefore, although there has not been reported a marked increase in the incidence of prostate cancer in the work reported earlier in the text [38, 40–43], further investigation, with appropriate follow-up time and tools, is needed before definitive conclusions can be drawn on the risk of prostate cancer in subjects with acromegaly.

## Medication for acromegaly and male sexual health

Among the drugs used for acromegaly, dopamine agonists could exert a positive effect on ED, as demonstrated in a randomized clinical trial in which cabergoline treatment was shown to be effective in treating psychogenic ED in patients with normal or mildly increased PRL levels [50]. In addition, cabergoline has proven effective in restore fertility in men with iperPRL due to micro- and macro-prolactinomas [51], but the effects of dopamine agonists on sexual health of men with acromegaly have not been studied. Conversely, the somatostatin analogue octreotide may have a negative effect on erectile function, as suggested by the inhibition of penile erection in rats [52] but there are currently no reports on humans. Similarly, octreotide transiently decreased sperm motility in stallions [53], but human data are lacking. Finally, the effects of pegvisomant on male sexual health are completely unexplored.

## Conclusions

The male sexual health represents an important element in acromegaly. Current evidence is relatively scarce but, given the impact of this pathology on the male sexual health and fertility, available data suggest paying great attention in clinical practice to this aspect of the pathology.

## Abbreviations

ED: Erectile Dysfunction
FSH: Follicle-Stimulating Hormone
GH: Growth Hormone
GHD: Growth Hormone Deficiency
GnRH: Gonadotropin-Releasing Hormone
IGF-1: Insulin-like Growth Factor-1
LH: Luteinizing Hormone
NO: Nitric Oxide
OGTT: Oral Glucose Tolerance Test
SHBG: Sex-Hormone Binding Globulin
SIEDY: Structured Interview on Erectile Dysfunction
